# Gene expression response under thermal stress in two Hawaiian corals is dominated by ploidy and genotype

**DOI:** 10.1002/ece3.70037

**Published:** 2024-07-24

**Authors:** Erin E. Chille, Timothy G. Stephens, Deeksha Misri, Emma L. Strand, Hollie M. Putnam, Debashish Bhattacharya

**Affiliations:** ^1^ Department of Biochemistry and Microbiology Rutgers University New Brunswick New Jersey USA; ^2^ Department of Genetics Rutgers University New Brunswick New Jersey USA; ^3^ Department of Biological Sciences University of Rhode Island Kingston Rhode Island USA; ^4^ Gloucester Marine Genomics Institute Gloucester Massachusetts USA

**Keywords:** asexual reproduction, clonal lineages, coral, gene expression, principal component analysis, triploidy

## Abstract

Transcriptome data are frequently used to investigate coral bleaching; however, the factors controlling gene expression in natural populations of these species are poorly understood. We studied two corals, *Montipora capitata* and *Pocillopora acuta*, that inhabit the sheltered Kāne'ohe Bay, Hawai'i. *M. capitata* colonies in the bay are outbreeding diploids, whereas *P. acuta* is a mixture of clonal diploids and triploids. Populations were sampled from six reefs and subjected to either control (no stress), thermal stress, pH stress, or combined pH and thermal stress treatments. RNA‐seq data were generated to test two competing hypotheses: (1) gene expression is largely independent of genotype, reflecting a shared treatment‐driven response (TDE) or, (2) genotype dominates gene expression, regardless of treatment (GDE). Our results strongly support the GDE model, even under severe stress. We suggest that post‐transcriptional processes (e.g., control of translation, protein turnover) modify the signal from the transcriptome, and may underlie the observed differences in coral bleaching sensitivity via the downstream proteome and metabolome.

## Graphical abstract

Reefs include coral species that differ vastly in lifestyle, reproductive strategy, stress resistance, and genetic background, with the interplay between these traits and their responses to climate change crucial to coral conservation. This study analyzed gene expression data from populations of two sympatric Hawaiian coral species, *Montipora capitata* and *Pocillopora acuta* that differ in genome ploidy. We tested whether gene expression patterns under thermal and pH stress comprise a set of shared stress‐responsive genes or reflect individual colony genotype.

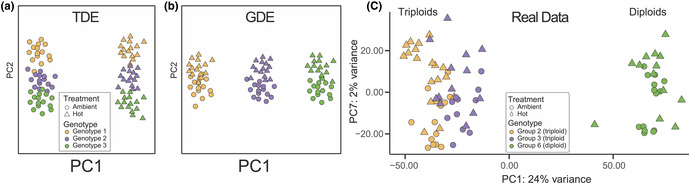

## 1 INTRODUCTION

Coral reef ecosystems are built on the nutritional symbiosis between scleractinian coral hosts and their single‐celled dinoflagellate (algal) endosymbionts in the family Symbiodiniaceae (Davies et al., 2023; LaJeunesse et al., 2018). These ecosystems are increasingly at risk from impacts caused by climate change, including ocean acidification (Orr et al., 2005), rising sea surface temperatures, as well as more frequent and intense marine heatwaves (Cheng et al., 2023). Thermal stress is a leading cause of coral mass mortality worldwide, resulting in dysbiosis between algal endosymbionts and the coral host, which can lead to the expulsion of algae from coral tissues (i.e., “bleaching”; Hughes et al., 2017). Ocean acidification, alternatively, places a chronic energetic strain on the coral host by increasing the cost of maintaining homeostatic processes under altered acid–base balance (Chille et al., 2022). A recent meta‐analysis shows that the interactive effects of ocean acidification and thermal stress on corals are primarily additive, resulting in increasing bleaching severity and mortality (Klein et al., 2022). However, the range of responses to these stressors varies greatly both within and between coral species (Baird & Marshall, 2002; Burgess et al., 2021; Guest et al., 2012, 2016; Mydlarz et al., 2010; Strand et al., 2024) and genotypes (Dixon et al., 2015, 2018; Poquita‐Du et al., 2020). Understanding the range of coral responses to warming and acidifying oceans is of critical importance (Eakin et al., 2022). Yet, this remains a challenging area of research because of the genetically diverse collection of organisms (cnidarian animal host, algal symbionts, prokaryotic microbiome, fungi and other eukaryotes, and viruses) that comprise the coral holobiont and contribute to its health and resilience (Cumming et al., 2023; National Academies of Sciences, Engineering, and Medicine, 2019; Veron, 2011).

Understanding the mechanisms underlying coral bleaching and the genomic basis for adaptive capacity has primarily been explored using transcriptome (RNA‐seq) data (Cziesielski et al., 2019; Kenkel & Wright, 2022; Young et al., 2023). However, these information‐rich datasets are often impacted by factors that can hinder straightforward interpretation, including host taxonomy and genotype, natural history, geographic origin, algal symbiont composition, and coral developmental stage (Ruggeri et al., 2022; Thomas et al., 2018). Understanding the factors that control gene expression, and therefore the bleaching response of corals in natural populations is crucial for inferring the correlation between these sources of information.

**FIGURE 1:**
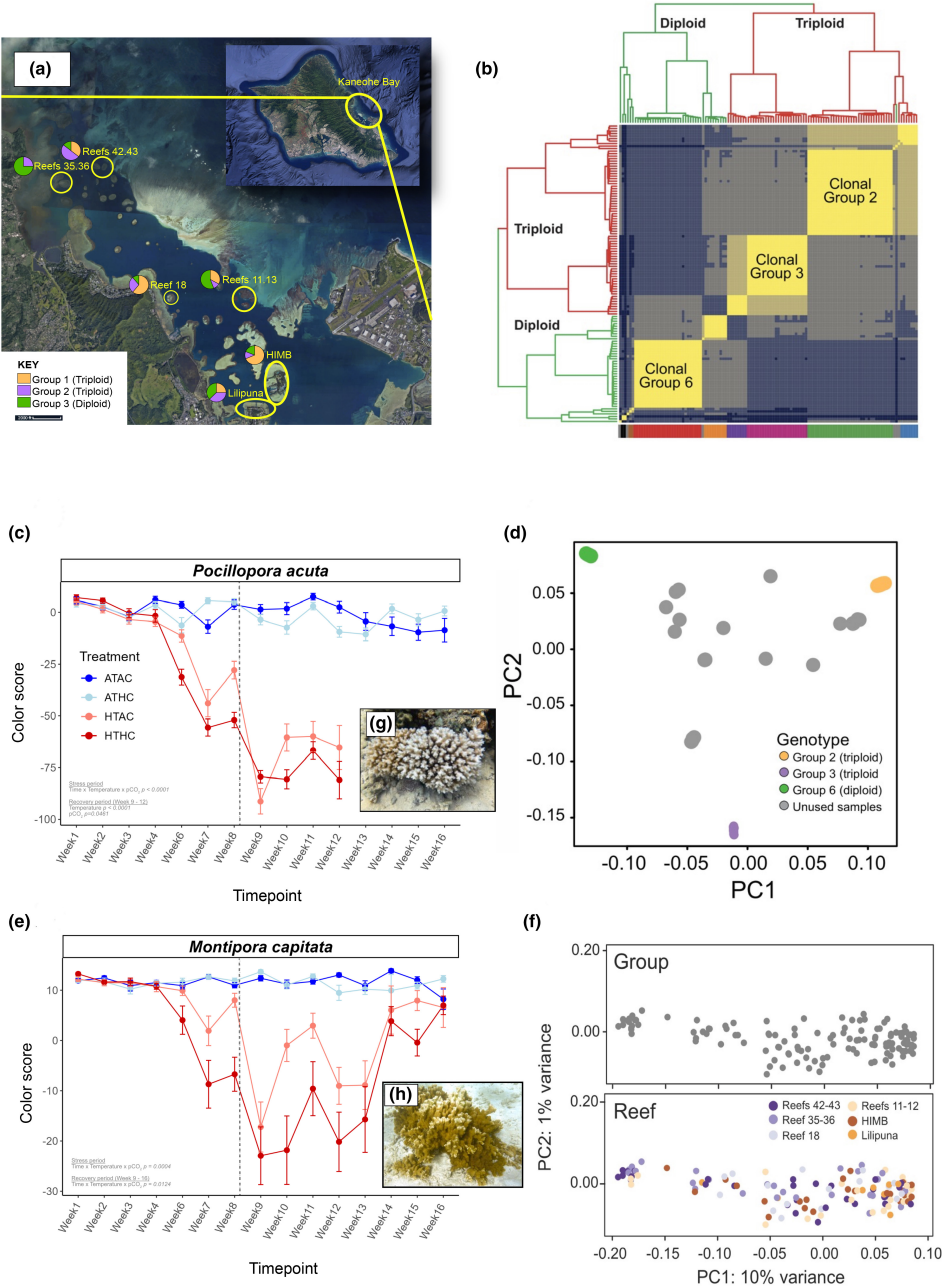
Experimental context for *Pocillopora acuta* and *Montipora capitata* RNA‐seq analyses. (a) Aerial image of Kāne'ohe Bay, O'ahu, Hawai'i with the six reefs where the coral samples were collected highlighted using yellow circles and labels (image modified from https://dlnr.hawaii.gov/). Proportional representation of the three studied clonal lineages of *P. acuta* (see key in bottom left) at each collection site is shown with the pie charts. These results do not provide evidence of genetic structure within and between the sampled reef areas. HIMB is the Hawai'i Institute of Marine Biology located on Moku o Lo'e. The legend for distance in feet is shown at the bottom left of the image. (b) Heatmap adapted from Stephens et al. (2023) showing *P. acuta* colony relatedness (based on Manichaikul et al., 2010) using all pair‐wise combinations of RNA‐seq data. The diploid and triploid groups are shown with green and red branches, respectively. The major clonal groups studied in the current paper are marked, with Clonal Groups 2 and 3 being triploid, and Clonal Group 6 being diploid. The eight major clonal groups that were identified by Stephens et al. (2023) are shown in different colors at the bottom of the heatmap. There is a single diploid *P. acuta* embedded among the triploids, which may be a case of reversion to the sexual state in this individual (for details, see Stephens et al., 2023). (c) Summary of color change results of *P. acuta* from Strand et al. (2024). Color change results indicate visual bleaching state and were quantified using color‐standardized photographs (Edmunds et al., 2003; Williams, Chiles, et al., 2021). The dashed vertical line indicates a return to ambient temperature between weeks 8 and 9. No results were collected from week 5. Significant effects from Strand et al. (2024) Type III ANOVA of linear mixed model outputs are listed in the bottom left of the panel. (d) PCA of *P. acuta* sample relatedness adapted from Stephens et al. (2023), whereby samples are colored by clonal group, showing the three analyzed in this study along with the other samples that were generated from Kāne'ohe Bay but were not analyzed here. A legend describing each color is shown in the bottom right of the panel. These plots are based on the covariance matrix produced by PCAngsd with estimated individual allele frequencies and show PC1 (17.54% variance explained) and PC2 (14.66% variance explained). (e) Summary of color change results of *M. capitata* from Strand et al. (2024). (f) PCA showing Stephens et al. (2023) results for *M. capitata* sample relatedness, with outliers removed, whereby samples are colored by clonal group (top) and reef (bottom). All samples in the clonal plot (top) are gray circles because no clonal groups were included in this analysis. (g) Two abutting colonies of the coral, *P. acuta* and (h) a large colony of *M. capitata*. Images taken by D. Bhattacharya.

To investigate the link between coral host natural history, genotype, and the abiotic stress response vis‐à‐vis gene expression, we compared RNA‐seq data from *Pocillopora acuta*, a species that spreads primarily clonally, to *Montipora capitata*, a species that is an obligate outbreeder (Stephens et al., 2023). We analyzed RNA‐seq data from Stephens et al. (2023) of 119 *P. acuta* corals from Kāne'ohe Bay, Hawai'i (Figure 1a,g), which were randomly distributed in tanks that simulated thermal and/or low pH stress conditions. Post‐hoc analysis of single‐nucleotide polymorphism (SNP) data from the studied *P. acuta* colonies identified eight main genotypes (encompassing 113/119 [94.96%] of the samples) that comprise distinct diploid and triploid clonal lineages that have arisen in the bay via asexual larval propagation or fragmentation (Figure 1b,d; Stephens et al., 2023). Therefore, we had the unique opportunity to assess intra‐ and inter‐genotype gene expression variation under abiotic stress, the effects of ploidy on this process, and to test the hypothesis that different genotypes of naturally derived clonal groups share gene regulation patterns for stress‐response genes. We also analyzed RNA‐seq data from 132 haphazardly sampled *M. capitata* colonies (Figure 1f,h), collected from the same reefs as the *P. acuta* samples, that comprise 130 distinct, diploid genotypes (Caruso et al., 2022; Stephens et al., 2023). The *M. capitata* colonies were subjected to the same abiotic stressors as *P. acuta*, allowing us to determine if a population‐wide core set of gene expression patterns exist, while controlling for genotype. Our work is buttressed by the availability of a chromosome‐level reference assembly for Hawaiian *M. capitata* (Stephens et al., 2022) and a high‐quality draft assembly of *P. acuta* from a triploid individual collected in Kāne'ohe Bay (Stephens et al., 2022) that acted as the reference genomes for these analyses, and physiological data (e.g., color score, chlorophyll content, symbiont cell density, photosynthetic rates, total antioxidant capacity) that were collected in parallel (from different samples) during this experiment (Strand et al., 2024). These authors provided evidence that the *M. capitata* holobiont is more thermally resistant (Figure 1e) and dominated by *Durusdinium* spp. algal endosymbionts, whereas *P. acuta* is relatively more thermally sensitive (Figure 1c) and dominated by *Cladocopium* spp. endosymbionts. Both species showed a significant physiological response to temperature, but not pCO_2_ treatment. Differences in host sensitivity to thermal stress appeared to be driven by elevated baseline photosynthetic rates in *P. acuta* and lower antioxidant capacity. Important for this study, because both species showed clear physiological responses to temperature, we were able to investigate the RNA‐seq data under two competing “strawman” models: (1) gene expression is largely independent of genotype, reflecting a shared response driven by physiological response to treatment (Treatment‐Driven Expression, TDE [e.g., Kenkel & Wright, 2022]) or, (2) genotype dominates gene expression, regardless of physiological response to treatment (Genotype‐Driven Expression, GDE).

## 2 METHODS

### 2.1 Coral collections and experimental setup

Coral samples (one ~5 × 5 cm^2^ fragment per colony) were collected from six reef areas ranging across the north‐to‐south span and fringing to patch reefs in Kāne'ohe Bay (Lilipuna Fringe: 21°25′45.9″ N 157°47′28.0″ W; HIMB: 21°26′09.8″ N 157°47′12.7″ W; Reef 18: 21°27′02.9″ N 157°48′40.1″ W; Reefs 11 and 13: 21°27′02.9″ N 157°47′41.8″ W; Reefs 35 and 36: 21°28′26.0″ N 157°50′01.2″ W; Reefs 42 and 43: 21°28′37.9″ N 157°49′36.8″ W) under Hawai'i Department of Aquatic Resources Special Activity Permit 2019‐60, between September 4–10, 2018 (Figure 1a). Fragments were hot‐glued to plugs and allowed to acclimate for ~14 days in outdoor mesocosm tanks and then randomly allocated to experimental tanks. R was used to randomly generate the distribution of fragments to the treatment tanks and the list of fragments to be collected from each tank on each sampling day.

### 2.2 Experimental timeline

Randomized coral fragments (with respect to site) were exposed to four experimental conditions: (1) Ambient Temperature, Ambient pCO_2_ (ATAC: 27.47°C ± 0.13; 8.03 pH ± 0.01, 396 μatm ±12), (2) Ambient Temperature, High pCO_2_ (ATHC: 27.46°C ± 0.12; 7.68 pH ± 0.014, 1045 μatm ±35), (3) High Temperature, Ambient pCO_2_ (HTAC: 29.37°C ± 0.06; 8.01 ± 0.01, 418 μatm ±13), and (4) High Temperature, High pCO_2_ (HTHC: 29.51°C ± 0.06; 7.64 ± 0.02, 1174 μatm ±55) for 2 months (22 September – 17 November 2018). At week 8 of the experiment, the high pCO_2_ treatments were ended and the high‐temperature treatments were gradually ramped down, returning to ambient temperature at week 9. Ambient conditions were then held for 2 months (18 November 2018–12 January 2019). During the four‐month stress exposure and recovery period, one fragment per species per tank (*n* = 3 per treatment) was sampled at random for each time point (0 h, 6 h, 12 h, 24 h, and 1, 2, 4, 6, 8, 12, and 16 weeks). Sampled fragments were placed in sterile Whirl‐Pak® bags, snap‐frozen in liquid nitrogen, and stored at −80°C.

### 2.3 Experimental tank setup

Outdoor mesocosm tanks (122 cm × 122 cm × 30 cm; 510 L volume) were randomly assigned a treatment condition (e.g., ATAC, ATHC, HTAC, HTHC; *n* = 3 replicate tanks treatment^−1^). Tanks were 60% shaded from full solar irradiance, and flow rates were set to allow a full tank turnover every 2 h. Flow rates were measured daily with a graduated cylinder and timer (84.36 ± 1.20 mL s^−1^; *n* = 826). In addition, photosynthetically active radiation (PAR) was measured continuously from the center of each tank with an Apex cosine corrected PAR Sensor (PMK, Neptune Systems, accuracy = ±5%) that was cross‐calibrated to a Li‐COR cosine corrected PAR sensor (LI‐193 spherical underwater quantum sensor, LI‐COR Biosciences). To reduce any potential effects of spatial position in the tank, the placement of each coral fragment within its assigned tank was changed weekly.

Temperature and pCO_2_ treatments were chosen to mimic the natural daily fluctuations in Kāne'ohe Bay and to reflect previous marine heatwaves (NOAA Moku o Lo'e Buoy data from September 2018, https://www.ndbc.noaa.gov/station_page.php?station=mokh1; Drupp et al., 2011) and projected pCO_2_ conditions for coastal embayments (Jury et al., 2013; Shaw et al., 2016). High‐temperature treatments fluctuated between ~29–30°C (+2°C above ambient temperature) to reflect previous marine heatwaves in Kāne'ohe Bay, while ambient temperature treatments reflected the natural conditions of Kāne'ohe Bay. Temperature was monitored continuously with an Apex Extended Life Temperature Probes (accuracy = ±0.05°C, Neptune Systems) and every 10 min using HOBO Water Temp Pro v2 temperature loggers (accuracy = ±0.21°C, resolution = 0.02°C, Onset Computer Corp). High‐temperature treatments were generated using submersible heaters (ProHeat D‐1500 Heater Controllers). High pCO_2_ conditions were maintained between 7.6–7.7 (pH NBS scale), while ambient conditions fluctuated between 7.9 and 8.0 (pH NBS scale). To generate a high pCO_2_ treatment, CO_2_ was delivered on‐demand to seawater via airlines plumbed into a Venturi injector (Forfuture‐go G1/2 Garden Irrigation Device Venturi Fertilizer Injector) from two 99.99% food‐grade CO_2_ cylinders. Temperature (°C), total scale pH, and salinity (PSU) were measured twice daily and total alkalinity and carbonate chemistry were measured twice weekly. For details of experimental setup, see Strand et al. (2024).

### 2.4 RNA extraction, sequencing, and read processing

RNA was extracted, sequenced, and processed from the coral fragments as described in Stephens et al. (2023). Briefly, RNA‐seq libraries were generated for 132 *M. capitata* and 119 *P. acuta* samples. The different number of samples sequenced for each species is explained by the higher mortality rate of *P. acuta* at later time points (week 16 was excluded from the RNA‐seq dataset) and issues with RNA extraction (one sample from the week 12 HTHC group, Table S1), possibly due to partial mortality. RNA was extracted using the Zymo Quick‐DNA/RNA Miniprep Plus Kit and sent to GENEWIZ (Azenta; https://www.genewiz.com) for library prep using the GENEWIZ “Standard RNA‐Seq” library preparation protocol for eukaryotic mRNA (i.e., poly‐A selection) and paired‐end sequencing (2 × 150) using the Illumina NovaSeq 6000 platform. Adapters and low‐quality regions were trimmed from reads using Cutadapt v2.9 (Martin, 2011; ‐‐nextseq‐trim 10 ‐‐minimum‐length 25 ‐a AGATCGGAAGAGCACACGTCTGAACTCCAGTCAC ‐A AGATCGGAAGAGCGTCGTGTAGGGAAAGAGTGTA). A second round of trimming with Cutadapt, using the output from the first round, was used to remove poly‐G regions from the 5′‐ends of the second read in each pair (‐G G{20} ‐e 0.0 ‐n 10 ‐‐minimum‐length 25; Table S2). Read quality was assessed at each stage using FastQC v0.11.7 (default parameters) and MultiQC v1.9 (Ewels et al., 2016). Cleaned reads were pseudo‐mapped to predicted protein‐coding genes from the *M. capitata* and *P. acuta* reference genomes (Stephens et al., 2022) and quantified for each sample using Salmon v1.6.0 (Patro et al., 2017; ‐‐validateMappings ‐‐seqBias ‐‐gcBias ‐‐libType ISR) with the associated reference genomes used as decoys during index building. For both species, the gene count matrix generated via Salmon was used for genome‐wide expression analysis. Genes with reads less than 3.33 counts per million (cpm) summed across all samples were removed from both matrices using the edgeR *cpm*() function (Chen et al., 2016). The threshold value of 3.33 cpm was calculated for the gene expression data based on the recommended formula: rowSums(cpm(gcount) > 10/L) >= *n*, where L is the minimum library size in millions of reads and *n* is the minimum number of replicates (*n* = 3). After pre‐filtering, data was normalized using the *calcNormFactors*() function to convert raw library sizes into effective (scaled) library sizes (Robinson & Oshlack, 2010).

### 2.5 Genome‐wide gene expression analysis

Principal component analysis (PCA) was used to investigate genome‐wide transcriptome patterns between diploid and triploid *P. acuta* and diploid *M. capitata* samples. To assess the relative influence of ploidy compared to genotype alone, two PCAs were done for *P. acuta*: one comparing 85 samples belonging to the largest triploid (Groups 2 and 3) and diploid (Group 6) genotypes, and one comparing the 58 samples belonging only to the largest triploid genotypes (Groups 2 and 3). A third PCA was done with the 85 Group 2, Group 3, and Group 6 *P. acuta* samples to validate whether mortality influenced global gene expression patterns during the later timepoints (see Supplementary Material for details). Only one PCA was performed using the 132 *M. capitata* samples to assess the influence of treatment on global gene expression in a model with high standing genetic variation. For all PCAs, the base R *prcomp*() function was used to calculate the principal components (PCs) from the cpm‐transformed gene count matrices. The total variation explained by each PC was calculated by dividing the squared standard deviation of each PC by the sum of all squared PCs. To assess the correlation between gene expression variation (as represented by each PC) and sample attributes (Treatment [temp + CO_2_], Temperature, CO_2_, Timepoint, Reef, Ploidy, and Genotype), Spearman correlation coefficients were calculated using the base R *cor*() function (Tables S3 and S4). The Kruskal–Wallis rank sum test was used to test the significance between a PC‐attribute pair when the Spearman correlation coefficient for that pair was >|.2| (Tables S5 and S6). Finally, PCA plots were generated using the R package ggplot2 (Wickham, 2011). All analyses were done in Rstudio (v.1.4.1106.0) with R version 4.0.5.

### 2.6 Differential gene expression analysis

The DESeq2 workflow (Love et al., 2014) was used to study differential gene expression between temperature treatments for *M. capitata* and *P. acuta*. To test the effect of genetic structure (i.e., *P. acuta* clonality compared to *M. capitata* high standing genetic diversity) on our ability to detect differential expression under thermal stress, we did differential gene expression analysis at the 1‐week time point. Here, each *M. capitata* fragment represented a different genotype, whereas the *P. acuta* genotypes were stratified across the two thermal treatments. That is, nearly every genotype was present in both treatments (Table S7). This allowed us to test and determine the relative impact of genotype versus treatment on differential gene expression, despite not controlling for genotype *a priori* in the original experimental design. For both species, log_2_FoldChange between HTAC and ATAC treatments was estimated at the 1‐week exposure timepoint (*n* = 3 per treatment [DESeqDataSetFromMatrix design = ~Treatment]).

To test for the effect of heat stress on gene expression in a single *P. acuta* genotype, differential expression analysis was also conducted on ambient and high‐temperature *P. acuta* samples from Group 2 at the 6‐week timepoint (DESeqDataSetFromMatrix design = ~Temperature). This time point was chosen because it was the only one with sufficient replicates from a single genotype to perform differential gene expression analysis. Due to the minor effects of pCO_2_ treatments on both the physiology (Strand et al., 2024) and global gene expression (see below), samples from both pCO_2_ conditions were included in this test to have an adequate number of replicates for each temperature (Ambient temperature: ATAC *n* = 1, ATHC *n* = 2; High temperature: HTAC *n* = 1, HTHC *n* = 3).

For all differential gene expression analyses, the Salmon‐generated count matrices were pre‐filtered to remove low‐coverage transcripts that may have been technical artifacts. The genefilter v1.74.1 *pOverA* function (Gentleman et al., 2020) was used to remove genes from the matrix wherein 50% of the samples had a count less than 10 (pOverA 0.5, 10). *p*‐Values were calculated using the Wald model and a log_2_FoldChange shrinkage of 1.5 was applied to prioritize genes with a larger effect size. Genes with a log_2_FoldChange > |2| and *p*‐adjusted value <.05 were considered differentially expressed (DE; Tables S8 and S9). DE gene putative function was assessed using blastx v2.14.1 with default parameters against the nr_cluster_seq database, entrez query “stony corals,” tax_id 6125 (Altschul et al., 1997). EggNOG functional annotations were also produced by EggNOG‐mapper v2.1.6 (parameters “‐‐pfam_realign denovo”; database retrieved 9th Dec. 2021) (Cantalapiedra et al., 2021) for all DE genes.

## 3 RESULTS AND DISCUSSION

Transcriptome data are frequently used to investigate the genomic basis for coral bleaching. However, the factors controlling gene expression in natural populations of coral species are poorly understood. Here, we investigated the effect of ploidy and population genetic structure on gene expression in two sympatric coral species, the predominantly clonal *P. acuta*, and the predominantly outbreeding *Montipora capitata* (Stephens et al., 2023). Our study took advantage of an experimental system in which physiological (Strand et al., 2024) and RNA‐seq data (Stephens et al., 2023) were collected from both coral species during stress (high temperature, high pCO_2_ [low pH], or both) and recovery. Given that both species exhibited a significant physiological response to thermal stress, but not to elevated pCO_2_ (Strand et al., 2024), we investigated whether the gene expression response reflects physiological response to treatment, or whether it is more strongly influenced by genotype. Here, we show that even under severe stress, genotype, not treatment (i.e., the GDE model), drives genome‐wide gene expression variation in these two species. We hypothesize that post‐transcriptional processes (e.g., control of translation, protein turnover) may underlie the inter‐genotype and specific differences in coral bleaching sensitivity via the downstream proteome and metabolome.

### 3.1 Effects of treatment and genotype on global gene expression patterns in *P. acuta*

For *P. acuta*, multiple genotypes representing extant Kāne'ohe Bay coral populations were exposed to abiotic stress (high temperature, high pCO_2_ [low pH], or both). RNA‐seq (Stephens et al., 2023) and physiological data (Strand et al., 2024) were collected from 119 diploid and triploid colonies of *P. acuta* (the majority of which were clonally derived). Strand et al. (2024) showed that *P. acuta* exhibited a severe physiological response to the elevated temperature, marked by a decrease in color score (shown here in Figure 1c), symbiont cell density, chlorophyll‐a, and respiration. Although physiological data were not taken from the same fragments as the RNA‐seq data, they were part of the same experiment, and because the fragments were randomly assigned (using R) to each dataset, the results from the physiology should be representative of the physiological state of the samples collected for RNA‐seq. Given the marked physiological response of the *P. acuta* exposed to elevated temperature, we asked whether a shared gene expression response to stress also exists within and between colonies from the three most abundant clonal genotypes (Groups 2, 3, and 6 [Figure 1d]; which accounted for 85 of the 119 samples; Figure 1b; Table 1). Stephens et al. (2023) showed that *P. acuta* clonal Groups 2 (34 colonies) and 3 (24 colonies) comprise distinct triploid lineages, whereas Group 6 (27 colonies) is a clonal diploid lineage (Figure 1d), and that the triploid lineages are diverged both from each other and from the diploid lineage (Figure 1b). We also expected limited confounding effects from collection site because these genotypes are distributed throughout Kāne'ohe Bay with no apparent fit to an isolation‐by‐distance model (Figure 1a, Stephens et al., 2023). Additionally, no significant gene expression response to mortality was observed (see Tables S10 and S11, Figure S2). Therefore, these RNA‐seq data (comprising 21,048 expressed genes that passed low abundance filtering) allowed us to test the fit of the gene expression data to the TDE and GDE hypotheses, and to disentangle the effects of ploidy and genotype on gene expression. Given the marginal impact of high pCO_2_ on *P. acuta* physiology when compared to thermal stress (Strand et al., 2024; Figure 1c), we assessed the impact of this stressor on gene expression using PCA. Only 1.04% cumulative variation in gene expression across all significantly correlated principal components (*p*‐value < .05; Table 2) was found for pCO_2_, therefore our downstream analysis (see “Differential gene expression during heat stress”) targeted only the temperature treatment and not pCO_2_ to test the competing TDE and GDE models. PCA was also used to test the fit of the competing TDE and GDE hypotheses.

**TABLE 1:** Number of *Pocillopora acuta* colonies in each group per treatment.

Treatment	Group 2 (triploid)	Group 3 (triploid)	Group 6 (diploid)	Grand total
Ambient	16	11	14	41
Hot	18	13	13	44
Total	34	24	27	85

**TABLE 2:** Summary of Spearman's correlation coefficients and significance of sample attributes and principal components for *Pocillopora acuta*.

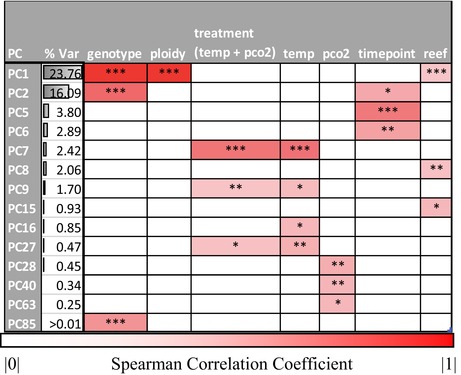

*Note*: Only principal components significantly associated with sample attributes and with Spearman's correlation >|.2| are shown. Full results are available in Tables S2 and S4. *p‐value <.05; **p‐value <.01; ***p‐value <.001.

**FIGURE 2:**
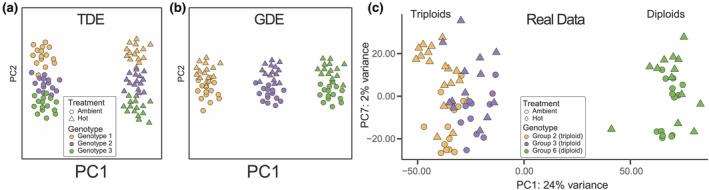
Testing Treatment‐Driven (TDE) and Genotype‐Driven (GDE) models of coral gene expression. (a) Expected outcome of PCA under extreme cases of TDE and (b) GDE (see text for details). The size of the PC1 and PC2 legends in these cases reflect the amount of variation (i.e., larger text = greater variation) being explained by these components. A legend describing sample genotype and treatment for the TDE and GDE models is shown in (a). (c) The actual results of PCA using the *Pocillopora acuta* gene expression data from the three clonal lineages, which strongly supports the GDE model. Here we used PC1 and PC7, which were the principal components most highly correlated with genotype and treatment, respectively (Table 2 and Table S4). The colors used to denote the two triploid and one diploid lineage are consistent across images.

Under TDE (Figure 2a), most of the variation that contributes to sample separation along theoretical PC1 would be attributable to treatment and most of the variation that contributes to sample separation along theoretical PC2, attributable to genotype. Under this scenario, *P. acuta* corals would share a conserved transcript‐based stress response that dominates the RNA‐seq data (see details below). Under GDE (Figure 2b), most of the variation along theoretical PC1 would be attributable to genotype and most of the variation along theoretical PC2, attributable to treatment. Under both models, the amount of variation captured by PC1 is significantly higher than PC2, represented in Figure 2a,b by the relative font sizes of the axis labels. Given these scenarios, where do the *P. acuta* data fit? As is apparent in Figure 2c, the genome‐wide gene expression data overwhelmingly support the GDE model. The PCA shows that ploidy and genotype are highly correlated with PC1 (Genotype: *r*_s_ = .9, *p*‐value = 1.32E‐15, Ploidy: *r*_s_ = −.81, *p*‐value = 1.46E‐13, Table 2), which accounts for a significant proportion (24%) of the variation in the data, whereas PC7, which is the highest order component significantly correlated (albeit weakly) with treatment (*r*_s_ = .54, *p*‐value = 16.43E‐07, Table 2), explains only 2.4% of data variation. The two triploid clonal genotypes (Groups 2 and 3) are positioned close together in the PCA, distinct from the diploids (Group 6). However, when considering only the two clonal triploid groups, Group 2 and Group 3 become greatly separated along PC1, which explains 28% of the variation in the data (Figure S1). This result demonstrates that coral colonies belonging to the same clonal group, despite originating from different reefs, have similar gene expression profiles, but the effect of ploidy (genome‐type) is stronger than genotype alone. Overall, these results show a weak genome‐wide shared transcriptomic stress response in *P. acuta*, which is consistent with the GDE model. That is, gene expression profiles observed in our experiment are primarily governed by genotype, as would be expected under GDE (Figure 2b).

Our results demonstrate that in *P. acuta* colonies, despite showing a significant physiological response to heat stress (i.e., bleaching [Figure 1c], Strand et al., 2024), gene expression is primarily driven by genotype. These results are consistent with previous genotype‐controlled gene expression studies in *Pocillopora acuta* (Poquita‐Du et al., 2020), *Acropora millepora* (Dixon et al., 2015, 2018), and *Acropora cervicornis* (Dilworth et al., 2024) that showed genotype explains a large proportion of variation in gene expression during thermal stress experiments (Dilworth et al., 2024; Dixon et al., 2015; Poquita‐Du et al., 2020) and reciprocal transplant experiments (Dixon et al., 2018). Therefore, we propose that the influence of genotype on gene expression is stronger than environment, that is, genotype‐by‐environment effects buffer the population‐wide transcriptomic response to stress. This leads to a central question: given that gene expression is presumed to respond to fluctuating environmental conditions, why does it not converge across genotypes under prolonged thermal stress, that in many cases leads to colony death in *Pocillopora* species (Baird & Marshall, 2002; Burgess et al., 2021; Guest et al., 2012, 2016; Strand et al., 2024)? One possible answer is that gene expression reflects past adaptive responses to local conditions that are not rapidly altered (e.g., “front‐loading”; Barshis et al., 2013). For this reason, downstream post‐transcriptional mechanisms such as differential access to the translation machinery, regulation of protein degradation, protein level buffering, and trans‐locus transcript abundance (Buccitelli & Selbach, 2020; Liu et al., 2016; Ponnala et al., 2014; Srivastava et al., 2022) may be critical to the coral stress response because they can be better tuned to local environmental conditions. These feedback systems (Arif et al., 2017; Kusnadi et al., 2022) will ultimately control enzyme abundance and activity (i.e., metabolite production that underlies the identified bleaching response).

Our system is atypical in the respect that we have controlled for genetic background by exploiting the natural reproductive system of *P. acuta* in Kāneʻohe Bay, which relies on clonal propagation, allowing both diploids and triploids to spread throughout the region. Kāneʻohe Bay fits the “everything is everywhere” model (Caruso et al., 2022; Stephens et al., 2023) (Figure 1a), resulting in a random distribution of coral larvae. Hence, we surmise that in this model system in which gene expression is controlled by genotype, post‐transcriptional regulatory mechanisms are likely critical to elicit the physiological response specific to different microhabitats. However, this pattern may not hold for other regions that show isolation‐by‐distance, whereby local adaptation is selectively advantageous for current and future generations. Understanding how these selective forces play out in different coral species and regions will likely be key to conservation efforts.

### 3.2 Effect of treatment on global gene expression patterns in *M. capitata*

**FIGURE 3:**
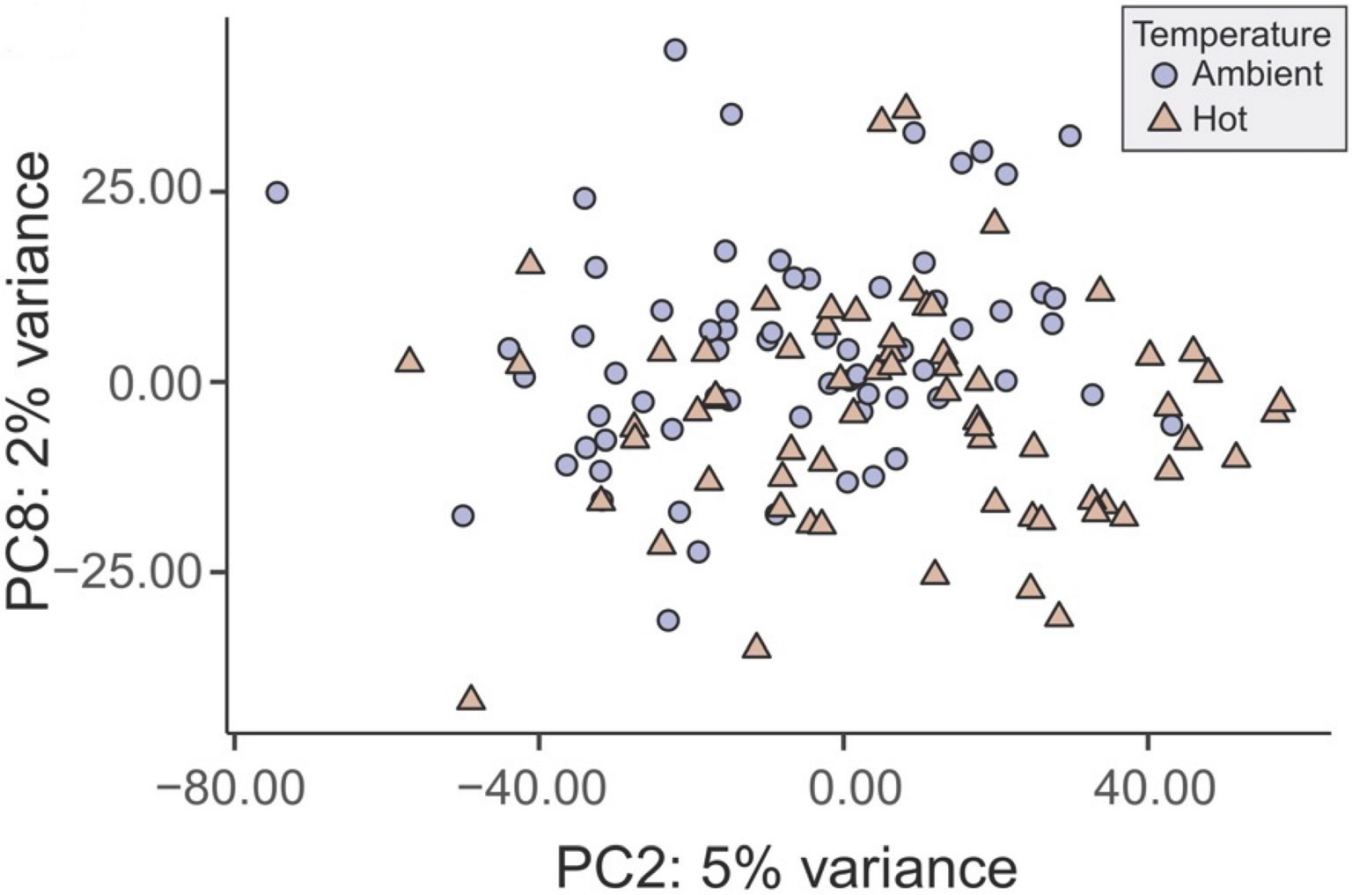
Results of PCA (showing the two PCs with the highest correlation with treatment) using the *Montipora capitata* gene expression data. Although the GDE model could not be explicitly tested due to the low prevalence of clonal colonies, the results do not support the TDE model. A legend describing sample treatment is shown in the top right of the panel.

To test the GDE model in a second coral species, we analyzed RNA‐seq data from the sympatric *M. capitata*, which relies on a different reproductive strategy to ensure survival. This species (Figure 1h) is relatively more resistant to thermal stress (Figure 1e; Strand et al., 2024, Williams, Pathmanathan, et al., 2021, Williams, Chiles, et al., 2021) than *P. acuta* (see Figure 1c) and is a hermaphroditic, mass‐spawning lineage. A strict outbreeder, *M. capitata* is essentially randomly distributed in Kāneʻohe Bay with respect to genotype (Figure 1f; Caruso et al., 2022; Stephens et al., 2023). Under the same experimental conditions used for *P. acuta*, RNA‐seq data were generated from 132 colonies and the 22,587 genes that passed expression filtering were analyzed as described above. Support for the GDE model could not be explicitly tested because, except for four samples that likely originated via local fragmentation, each colony comprised a distinct genotype (Stephens et al., 2023). Therefore, we assessed fit to the TDE model, which these data do not support. Spearman correlations between the principal coordinates and sample attributes show very weak correlation of temperature with PC2 (*r*_s_ = .28, *p*‐value = .0014) and PC8 (*r*_s_ = −.28, *p*‐value = .0012), which explain 5% and 2% of variation in the gene expression data, respectively (Figure 3; Table S6), suggesting that stress treatment explains very little variation in these RNA‐seq data and does not fit the TDE model.

### 3.3 Differential gene expression during heat stress in *P. acuta* and *M. capitata*

Under GDE, we expect a minor, albeit important treatment‐driven expression pattern. To determine which set of genes are driving the small difference between ambient and heat‐stressed corals, we performed differential gene expression analysis. *M. capitata*, which is comprised of distinct genotypes, provided a model in which we could search for the existence of a core set of population‐wide genes that are differentially regulated under stress in genetically heterogeneous colonies. Analysis conducted between heat‐stressed (HTAC) and non‐stressed (ATAC) *M. capitata* samples at 1‐week exposure revealed 31 differentially expressed (DE) genes out of the 18,225 that passed low‐abundance filtering (Table S8). Among the top over‐expressed genes under heat stress were paraspeckle component 1‐like isoform X2 (log_2_FoldChange 11.4, *p*_adj_ < .01), which is associated with the formation of stress granules (An et al., 2019), and sacsin‐like (log_2_FoldChange 2.2; *p*_adj_ < .01), which is a co‐chaperone of Hsp70 (Parfitt et al., 2009; Takahashi‐Kariyazono & Terai, 2021). These 31 genes likely represent a core set of genes that modulate the transcriptome‐based bleaching response in *M. capitata*.

Given the number of clones identified from *P. acuta*, we were able to test whether genotypic effects abolish or reinforce the existence of a population‐wide set of stress‐responsive genes. In contrast to *M. capitata*, analysis conducted between HTAC and ATAC *P. acuta* samples at 1‐week exposure revealed no DE genes. These results are likely explained by the fact that genotypic variation in the expression of stress‐responsive genes exceeds treatment‐driven variation, because samples belonging to the same genotypes were present in both conditions. However, when testing for treatment effects between heat‐stressed (pooled HTAC+HTHC) and non‐heat stressed (pooled ATAC+ATHC) within a single genotype of *P. acuta* (Group 2, week 6), treatment‐driven expression was detected for 78 genes (out of the 17,981 that passed low‐abundance filtering; Table S9). Several of these DE genes in Group 2 have been identified in other coral heat‐stress studies, including gamma‐crystallin M1‐like (log_2_FoldChange 10.2, *p*_adj_ < .01; Mayfield et al., 2018), 4‐hydroxyphenylpyruvate dioxygenase‐like isoform X2 (log_2_FoldChange 2.2, *p*_adj_ < .01; Ip et al., 2022) and putative ammonium transporter 1 (log_2_FoldChange − 4.6, *p*_adj_ < .01; Cleves, Krediet, et al., 2020; Williams, Pathmanathan, et al., 2021; Williams, Chiles, et al., 2021). Overall, these results show that whereas genome‐wide expression is driven primarily by genotype, treatment‐driven expression also occurs, but is dominated by genotypic variation. Therefore, the need to control and test for genotype and genotype‐by‐treatment interactions in future coral heat stress studies is paramount.

## 4 CONCLUSIONS

Our findings broaden understanding of coral gene expression and demonstrate that two different coral species have divergent responses to stress vis‐à‐vis gene expression. *P. acuta* and *M. capitata* in Kāneʻohe Bay do not share a broadly conserved transcriptome response to stress across genotypes, which does not support the TDE model (Cziesielski et al., 2019) as defined here (Figure 2a). Although we are unable to test for genotype‐by‐treatment effects, we find that genotype and ploidy drive genome‐wide gene expression variation much more than treatment (i.e., the GDE model, Figure 2b,c) in these two species. These results provide a novel perspective, yet there are many coral genes whose expression has been shown to be stress‐responsive, including those that are “front‐loaded” and may confer physiological resilience under frequent stress exposure (Barshis et al., 2013; Fifer et al., 2021). Therefore, RNA‐seq data is undoubtedly informative about coral stress responses (Cleves, Shumaker, et al., 2020).

The impact of genotype on gene expression is also not surprising, and likely reflects local adaptation of the parental lines, likely prior to transport to Kāneʻohe Bay (Dixon et al., 2015; Kenkel & Matz, 2016). Coral genotype in our model Hawaiian species clearly modulates the transcriptomic response to stress despite a convergence in observed stress response (Figure 1c,d). Whereas the observed stress response could be due to genotype‐by‐treatment interactions, that is, every genotype has a different path to the same phenotype, an alternative but not mutually exclusive hypothesis is that post‐transcriptional processes may modify the signal from the transcriptome to stress‐related outcomes that we have observed. Proteome and metabolome data (e.g., Camp et al., 2022; Pei et al., 2022; Williams, Chiles, et al., 2021) may therefore be better proxies for coral physiology. This idea remains to be tested in future studies that target a range of different coral species from different geographic origins.

Given these observations, our results underline the importance of characterizing genetic structure and reproductive behavior of corals to allow informed interpretation of “omics” data. The underpinnings of coral resistance and resilience involve not only algal symbiont and prokaryotic microbiome contributions but also fundamentally reflect host genotype(s) and their provenance (Dixon et al., 2015; Fuller et al., 2020). This is particularly pertinent with the acceleration of the search for transferrable mechanisms for use as human interventions to assist coral resistance and resilience and maintain these invaluable coral reef ecosystems under increasing global change.

## AUTHOR CONTRIBUTIONS

**Erin E. Chille:** Conceptualization (equal); methodology (equal); writing – original draft (equal); writing – review and editing (equal). **Timothy G. Stephens:** Conceptualization (equal); methodology (equal); writing – review and editing (equal). **Deeksha Misri:** Methodology (equal); writing – review and editing (equal). **Emma L. Strand:** Methodology (equal); writing – review and editing (equal). **Hollie M. Putnam:** Methodology (equal); supervision (equal); writing – review and editing (equal). **Debashish Bhattacharya:** Conceptualization (equal); supervision (equal); writing – original draft (equal); writing – review and editing (equal).

## CONFLICT OF INTEREST STATEMENT

The authors declare no conflicts of interest.

## Supporting information


Appendix S1.

## Data Availability

The RNA sequencing data are available from NCBI's SRA repository (BioProject: PRJNA731596). Genome assemblies and predicted genes are available from http://cyanophora.rutgers.edu/montipora/ (Version 3) and http://cyanophora.rutgers.edu/Pocillopora_acuta/ (Version 2). Read processing scripts are available at https://doi.org/10.5281/zenodo.8077955. Data, metadata, scripts, and output from all gene expression analyses are available at https://doi.org/10.5281/zenodo.10652225.
