# Supplementary material for: Gene expression response under thermal stress in two Hawaiian corals is dominated by ploidy and genotype
Source: Ecol Evol. 2024 Jul 24;14(7):e70037. doi: 10.1002/ece3.70037 (PMC11268936; doi:10.1002/ece3.70037)
Supplement: Supplementary file 1 — Appendix S1. [file ECE3-14-e70037-s001.zip › Chille_etal_2024_EcolEvol_R1_Supplementary_Material.docx]

**Gene expression response under thermal stress in two Hawaiian corals is dominated by ploidy and genotype**

Erin E. Chille, Timothy G. Stephens, Deeksha Misri, Emma L. Strand, Hollie M. Putnam, and Debashish Bhattacharya

##

## Table of Contents

[Supplementary Methods and Results 1](#_Toc168254447)

[Assessing for batch effects in *P. acuta* due to mortality in recovery period 1](#_Toc168254448)

[Supplementary Figures 2](#_Toc168254449)

[Supplementary Tables 3](#_Toc168254450)

[References 5](#_Toc168254451)

## Supplementary Methods and Results

### Assessing for batch effects in P. acuta due to mortality in recovery period

Due to mortality observed during the recovery period of the experiment, resulting in the loss of one fragment from the week 12 timepoint (**Table S1**), downstream analysis was conducted to determine whether mortality influenced global gene expression. PC5 and PC6 from the PCA involving the 85 samples from clonal Group 2, Group 3, and Group 6, (see “Genome-wide gene expression analysis” in the Methods), were further analyzed because they were significantly correlated with timepoint with no other covariates (**Table 2**). A PCA plot was generated for PC5 and PC6 as described under “Genome-wide gene expression analysis” in the Methods. To evaluate shifts in global gene expression through time, the mean eigenvalue for each timepoint in each temperature treatment was overlaid on top of the eigenvalues for each sample (**Fig. S2**). This analysis showed that on average, global gene expression appeared to return to baseline values before the start of the experiment. Spearman correlation coefficients for PC5 and PC6 were calculated using the base R *cor()* function to assess correlation of eigenvalue to genotype, timepoint, and combined genotype_timepoint. Then, Dunn Kruskal-Wallis multiple comparison tests (Dunn, 1961) were performed using the FSA package *dunnTest* function in R for the ambient and high temperature treatments to determine whether there was a correlation between individual timepoints with PC5 or PC6. Adjusted *p*-values for the Dunn test were calculated using the Benjamini-Hochberg method. Spearman analysis showed that PC5 and PC6 were both significantly correlated with timepoint and timepoint_genotype, but not with genotype alone (**Table S10**). However, the eigenvalues for PC5 and PC6 gene expression did not show any significant differences (*p*-adjusted ≥ 0.05) between week 12 and other timepoints in the hot treatment (**Table S11**). This, and because prior to the beginning of the experiment, a random number generator was used to determine which fragments to collect at each time point, we do not expect that mortality of more thermally-sensitive genotypes significantly affected the outcomes of our global gene expression patterns or differential expression analysis.

## Supplementary Figures

**Figure S1.** **Principal components analysis using the *P. acuta* gene expression data from the two triploid clonal lineages (58 samples).** Here we used PC1 and PC7, which were the principal components most highly correlated with genotype and treatment, respectively (Table 2, Table S4) from the analysis using 85 samples across the largest two triploid and one diploid clonal groups (**Fig. 2c**). The colors used to denote the two triploid lineages are consistent across images.


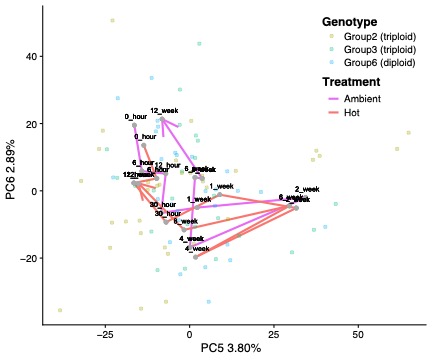


**Figure S2. Principal components analysis using the *P. acuta* gene expression data** from the two triploid and one diploid lineages (85 samples) which strongly supports the GDE model. Here we used PC5 and PC6, which were the principal components most highly correlated with timepoint and without covariation with other factors (Table 2, Table S4). The colors used to denote three clonal lineages are not consistent with Figures 2 and S1, this was done to place the focus on the effect of timepoint rather than genotype. The average eigenvalue for each timepoint along PC5 and PC6, calculated separately for the ambient (pink) and hot treatments (red), are overlayed on top of the sample timepoints in grey.

## Supplementary Tables

**Table S1**. Summary of the number of RNA-seq samples across timepoints. Timepoints 10 and 11 are during the recovery period of the experiment. Timepoint 11 was the only timepoint with fewer than three samples collected for a treatment (denoted in red).

**Table S2.** Statistics for the raw and quality-controlled RNA-seq data generated from each sample.

**Table S3.** Table of Spearman correlation coefficients between principal components scores for each sample, computed from cpm-transform counts of 21,048 genes in 85 *P. acuta* samples, and the sample's attributes, including treatment (combined temperature and CO2), temperature, CO_2_, timepoint, and reef.

**Table S4.** Table of Spearman correlation coefficients between principal components scores for each sample, computed from cpm-transform counts of 22,587 genes in 132 *M. capitata* samples, and the sample's attributes, including treatment (combined temperature and CO_2_), temperature, CO2, timepoint, reef, and tank.

**Table S5.** Table of Kruskal-Wallis rank sum test results for *P. acuta* to test for significance of Spearman Correlation Coefficients >|0.2| as presented in Table S3, including principal component, treatment (combined temperature and CO_2_), temperature, CO_2_, timepoint, and reef.

**Table S6.** Table of Kruskal-Wallis rank sum test results for *M. capitata* to test for significance of Spearman Correlation Coefficients >|0.2| as presented in Table S4, including principal component, treatment (combined temperature and CO_2_), temperature, CO_2_, timepoint, reef, and tank.

**Table S7.** Summary of the number of RNA-seq *P. acuta* genotypes in the Ambient temperature, Ambient CO_2_ (ATAC) and High temperature, Ambient CO_2_ (HTAC) and across the experimental timepoints. Timepoint 1_week is highlighted in green because it was chosen for differential expression analysis, as two of the four genotypes sampled at that time were present in both the ATAC and HTAC treatments.

**Table S8.** Differential gene expression results for *M. capitata* samples exposed to 27.47 °C ± 0.13 (Ambient temperature) and 29.37 °C ± 0.06 (High temperature) at 1-week exposure. log2FoldChange results are reported for high temperature relative to ambient condition samples. Sample annotations were obtained from blastx against the nr_cluster_seq database, entrez_query "stony corals", tax_id 6125. EggNog functional annotations were retrieved from Stephens et al. (2023).

**Table S9.** Differential gene expression results for *P. acuta* Group 2 (triploid) samples exposed to 27.47 °C ± 0.13 (Ambient temperature) and 29.37 °C ± 0.06 (High temperature) at 6 weeks of exposure. log2FoldChange results are reported for high temperature relative to ambient condition samples. Sample annotations were obtained from blastx against the nr_cluster_seq database, entrez_query "stony corals", tax_id 6125. EggNog functional annotations were retrieved from Stephens et al. (2023).

**Table S10.** Table of Kruskal-Wallis rank sum test results for *P. acuta* to test for significance of Spearman Correlation Coefficients as presented in **Table S3**, including only principal components 5 (PC5) and 6 (PC6), timepoint, genotype, and combined genotype_treatment.

**Table S11.** Table of Dunn (1964) Kruskal-Wallis multiple comparison results for *P. acuta* to test for significance of Spearman Correlation Coefficients (**Table S10**) between principal components PC5 and PC6 and experimental timepoints. Results were calculated for samples in ambient and high temperature treatments separately. Dunn *p*-values were adjusted with the Benjamini-Hochberg method. Results are ordered by P.adj (*p*-adjusted) from small to large, with the dashed line showing indicating comparisons with a significant P.adj (0.05 ≥ *p*-adjusted). Signiant comparisons involving the 12_week timepoint, during which some mortality was observed among experimental fragments in the hot treatment, are highlighted in green.

## References

Dunn, O. J. 1961. Multiple Comparisons among Means. J. Am. Stat. Assoc. **56**: 52–64. doi:10.1080/01621459.1961.10482090
