# Supplementary figures and images for: Gene expression response under thermal stress in two Hawaiian corals is dominated by ploidy and genotype
Source: Ecol Evol. 2024 Jul 24;14(7):e70037. doi: 10.1002/ece3.70037 (PMC11268936; doi:10.1002/ece3.70037)

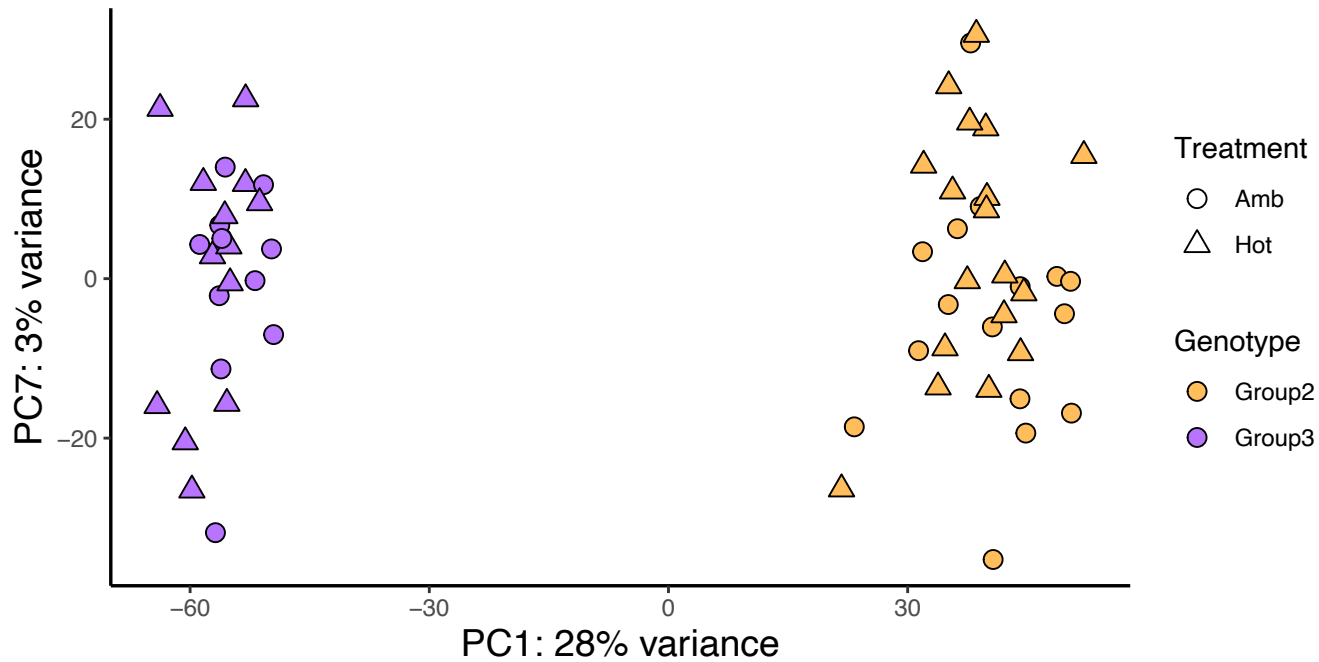

Supplement: Supplementary file 1 — Appendix S1. [file ECE3-14-e70037-s001.zip › Figure_S1.pdf]

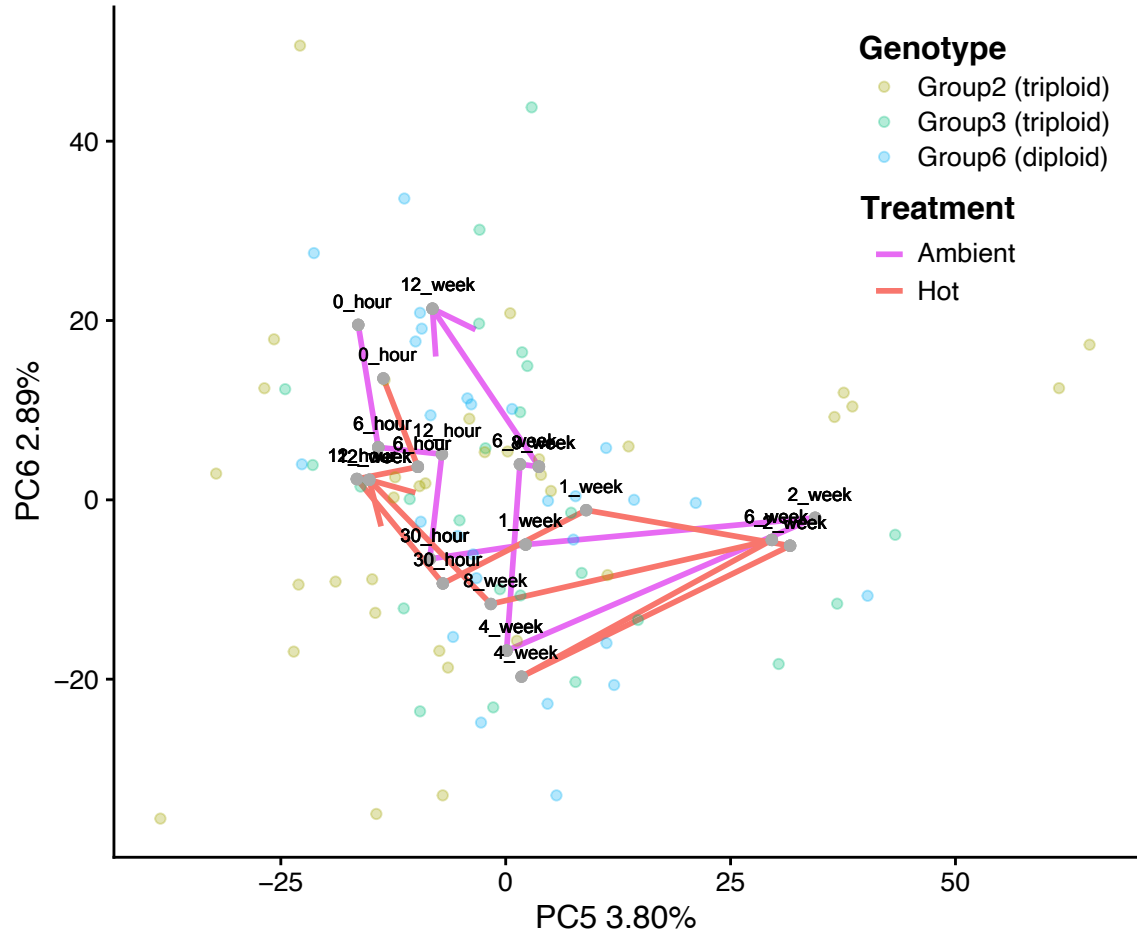

Supplement: Supplementary file 1 — Appendix S1. [file ECE3-14-e70037-s001.zip › Figure_S2.pdf]
